# Retinal Sensitivity and Retinal Perfusion in Diabetic Retinopathy

**DOI:** 10.1001/jamaophthalmol.2025.3980

**Published:** 2025-10-30

**Authors:** Jennifer A. Hamilton-Perais, David M. Wright, Amelia Lim, Ajay Mohite, Gerard Reid, Pearse Hillis, Cora Sheeran, Noemi Lois

**Affiliations:** 1 The Wellcome-Wolfson Institute for Experimental Medicine, Queen’s University Belfast, Belfast, Northern Ireland, United Kingdom; 2 The Centre for Public Health Queen’s University Belfast, Belfast, Northern Ireland, United Kingdom; 3 The Gleneagles Hospital Penang, Penang, Malaysia; 4 The Belfast Health and Social Care Trust, Belfast, Northern Ireland, United Kingdom; 5 The Royal Free London Foundation Trust, London, United Kingdom

## Abstract

**Importance:**

Retinal capillary nonperfusion seems crucial in the pathogenesis of sight-threatening diabetic retinopathy (DR); currently, no treatment prevents or reverts it.

**Objective:**

To further the understanding of the association between retinal capillary nonperfusion and sensitivity in DR.

**Design, Setting, and Participants:**

This prospective, longitudinal cohort study was conducted from April 18, 2018, to September 9, 2024, at a single center in the UK. Participants were followed up for up to 2 years; outcome assessors were masked. Adults (aged ≥18 years) with moderate or severe to very severe nonproliferative or proliferative DR with less than high-risk characteristics; at least 1 eye naive to treatment; no other retinal disorders; who were able to provide informed consent; and who were willing undergo retinal imaging were eligible for inclusion. Data analysis was performed from September 2024 to April 2025.

**Main Outcomes and Measures:**

The primary outcome was the association between retinal sensitivity (110° projection perimetry) and retinal perfusion (ultra-widefield angiography) at baseline and changes at 1 and 2 years in the study eye.

**Results:**

Of 66 people approached, 50 were eligible and recruited, and 44 individuals with at least 1 perimetric examination were included. Mean (SD) participant age was 52.1 (12.2) years, and 13 participants (29%) were female. Median hemoglobin A_1c_ was 75.5 mmol/mol (9.1% of total hemoglobin [to convert from percentage of total hemoglobin to proportion of total hemoglobin, multiply by 0.01]); mean (SD) best-corrected visual acuity letter score was 85.7 (4.7) (Snellen equivalent, 20/20). Mean retinal sensitivity deficit at baseline was associated with perfusion status, with larger deficits in nonperfused areas (n = 354; 11.8 dBs; 95% CI, 10.8-12.8) compared to perfused areas (n = 2092; 6.6 dB; 95% CI, 5.1-8.2; *P* < .001). Only age correlated positively with sensitivity deficit (estimate, 0.2; 95% CI, 0.1-0.3; *P* = .006). A deficit of 5 dB or greater occurred in 711 of 2092 (34%) perfused areas; 105 of 354 (30%) nonperfused areas had normal sensitivity. Rates of sensitivity deficit change in perfused and nonperfused areas from baseline to 1 year were −0.20 dB/mo (95% CI, −0.24 to −0.16) and −0.28 dB/mo (95% CI, −0.41 to −0.15) (perfused vs nonperfused, *P* = .22), respectively (1464 areas); from baseline to 2 years, rates were −0.16 dB/mo (95% CI, −0.20 to −0.12) and −0.34 dB/mo (95% CI, −0.47 to −0.21) (perfused vs nonperfused, *P* = .007), respectively (542 areas).

**Conclusions and Relevance:**

In this longitudinal cohort study, although retinal capillary perfusion status was associated with function, sensitivity loss occurred in some perfused areas and normal function in some nonperfused areas; sensitivity deficit decreased over time (approximately 45% in the first year) despite poor glycemic control and high DR grades. These findings should be considered for the management of people with DR and the design of clinical trials.

## Key Points

**Question:**

Is there an association between retinal capillary nonperfusion and retinal sensitivity in people with higher stages of diabetic retinopathy, and how does this association change over time?

**Findings:**

In this longitudinal cohort study including people with moderate nonproliferative through less than high-risk proliferative diabetic retinopathy (n = 44), retinal capillary nonperfusion was associated with reduced retinal sensitivity, with a reduction in functional deficits occurring in both perfused and nonperfused retinal areas during the follow-up of up to 2 years.

**Meaning:**

These findings further the understanding of diabetic retinopathy and should be considered in the design of interventional trials for capillary nonperfusion.

## Teaser

This longitudinal cohort study conducted in the UK explores the association between retinal capillary nonperfusion and sensitivity in diabetic retinopathy.

## Introduction

Retinal capillary nonperfusion (CNP) is crucial in the pathogenesis of diabetic retinopathy (DR)^1^ and is a main driver of proliferative DR (PDR) and its complications, namely tractional retinal detachment, vitreous hemorrhage, rubeosis iridis, and neovascular glaucoma. CNP is also the defining feature of diabetic macular ischemia. Despite being a nearly universal event in DR,^2^ for unclear reasons, its resulting aforementioned complications occur relatively infrequently.^3,4,5^ CNP may not be evident unless it is revealed by fundus fluorescein angiography (FFA) or optical coherence tomography angiography (OCTA). To date, there is no therapy to prevent its occurrence or to revert it (ie, achieve revascularization). Diabetes UK has recognized CNP as a research priority to reduce sight loss.^6^

Relatively few previously conducted studies have investigated the association between retinal sensitivity and retinal perfusion in DR, testing areas of the retina of up to 60°.^7,8,9,10,11,12,13,14,15,16^ Most studies were cross-sectional^7,8,9,10,11,12,13,14,15,16,17^ and small (13-25 patients)^8,10,11,12,13,14,16^; some included previously treated patients.^13,14,17^ The few longitudinal studies undertaken, with a follow-up of up to 1 year, evaluated the association between perfusion and function only in the center of the macula^18^ or only at the fovea.^19^ The only prospective study investigating point-to-point perfusion sensitivity tested the fovea only.^19^

Ultra-widefield (UWF) fundus imaging has enabled a comprehensive examination of retinal CNP.^20^ Advances in automated perimetry through the introduction of full-field projection perimeters have provided more reliable and repeatable examinations of retinal function.^21^ The combination of these modalities facilitates the evaluation of more precise and extensive point-to-point structural-functional relationships throughout the retina.

With this background in mind, we investigated herein the effect of CNP on retinal sensitivity in a prospective longitudinal cohort study of people living with diabetes and DR.

## Methods

### Study Design and Eligibility Criteria

This study was part of a larger prospective, observational, longitudinal cohort study conducted at the Belfast Health and Social Care Trust, Northern Ireland, UK, between April 18, 2018, and September 9, 2024, for which approval was obtained (14/NI/0076). It was conducted according to the principles originating from the Declaration of Helsinki and reported following the Strengthening the Reporting of Observational Studies in Epidemiology (STROBE) reporting guidelines.

Adults (aged ≥18 years) with type 1 or 2 diabetes and moderate or severe to very severe nonproliferative DR, or PDR with less than high-risk characteristics (eFigure 1 in Supplement 1), were eligible if they had at least 1 eye naive to treatment, no other retinal disorders, and were able to provide informed consent and undergo retinal imaging. Grading of retinopathy was undertaken by an experienced clinician based on fundus examination and UWF images (pseudocolor and FFA). Patients were consecutively approached; written informed consent was obtained prior to performing study procedures. Participants received no stipend.

### Outcome Measures

Demographics and medical history were completed at baseline and reviewed at each visit. Participants had hemoglobin A_1c_ (HbA_1c_) testing, best-corrected visual acuity (BCVA) by an optometrist (J.P.), and clinical examination by an ophthalmologist (A.L., A.M., G.R., or N.L.) at baseline and at months 6, 12, 18, and 24 (±30 days). The eye with the more severe DR grading^22^ or, in people with 1 eye previously treated with laser panretinal photocoagulation, the untreated eye (ie, the eye naive to treatment), was the study eye. If both eyes had identical grading, the right eye was chosen arbitrarily as the study eye. For the analysis presented herein, outcome measures included retinal sensitivity and retinal perfusion at baseline and at 1 and 2 years.

Static automated perimetric threshold examinations (MonCvONE perimeter, software version 2023J [Metrovision]) were conducted by the same optometrist (J.P.), monocularly, in darkened room conditions, generating 57 retinal sensitivity deficit values across a 110° field. Deficit values represent the difference between threshold sensitivity (minimal luminance detectable) measured at a particular retinal point and that of age-matched healthy individuals. These normative values were based on 160 individuals of different ages (<40 years: 119 individuals; 40-60 years: 30; >60 years: 11) (data from Metrovision).^23^

Perimetry was introduced once the larger study (which has other objectives) had commenced; thus, perimetry was not performed in all participants at all visits (see Results). There was no selection bias introduced, as all participants received perimetry upon joining the study once the perimeter became available (ie, all participants had a baseline perimetry, some had a 1-year perimetry [1 year following baseline], and a smaller group had a 2-years perimetry [2 years following baseline]).

Refractive correction for near was provided for participants with presbyopia when assessing the central field and removed for the peripheral examination to prevent obscuration of stimuli by the trial frame. A live video camera displaying the patient’s eye ensured its alignment with a fixation target throughout the examination.

On completion of functional tests and following pupillary dilatation, pseudocolor and UWF-FFA images were obtained (California [Optos]).^24^ Eyes were held open during image acquisition to minimize eyelid or eyelash artifacts; superior and inferior steered images were acquired to maximize imaged area.

### Grading of UWF-FFA Images

To grade CNP, the clearest early venous laminar flow phase frame with the most extensive field of view was selected. Regions of interest were demarcated and measured (in millimeters squared) using Optos Advance analyzer software, version 5.1,^25^ which automatically corrects for inherent peripheral distortion resulting from the spherical retinal surface being projected 2-dimensionally. Image-enhancing tools were used to optimize images’ contrast, clarity, and visibility. If regions of the image could not be visualized due to blurriness or transient opacities (eg, vitreous floaters), earlier and/or later frames were additionally evaluated. Pseudocolor images were cross-referenced to ensure areas graded as nonperfused did not correspond to other lesions (eg, hemorrhages) blocking fluorescence. The region-of-interest tool was used to demarcate the total retinal area imaged, with ungradable areas resulting from artifacts and nonperfused areas. Areas of nonperfusion were identified by their hypofluorescence, most often surrounded by pruned capillaries and microaneurysms, contrasting with the healthy perfused retina. Trained graders (P.H., C.S.) graded all angiograms masked to clinical information and functional data; these were reviewed by an experienced second grader (J.P.), consulting with an ophthalmologist (N.L.) when discrepancies or uncertainties occurred.

Baseline angiograms were graded first, followed by 1- and 2-year angiograms. The area of retina imaged, the area with artifacts, and nonperfused regions were totaled for each eye. The retinal ischemic index (RII) per eye was calculated as follows: RII = Total area of CNP (mm^2^)/[Total retinal area imaged − ungradable areas (mm^2^)] × 100.

To determine the reproducibility of measures of RII, gradings were repeated, masked, in 10 unselected consecutive eyes 3 months after the original gradings.

All UWF-FFA areas with a change in grading (ie, perfused to nonperfused or nonperfused to perfused) at any time point (ie, baseline to 1 year or baseline to 2 years) were reviewed again, masked to clinical and perimetric findings, to ensure changes were genuine, eliminating potential grading errors. For this, UWF-FFAs were compared on dual screens, side by side, and areas of progression or reperfusion were verified or eliminated.

### Combining Graded Angiograms With Perimetric Data

**Figure 1.**
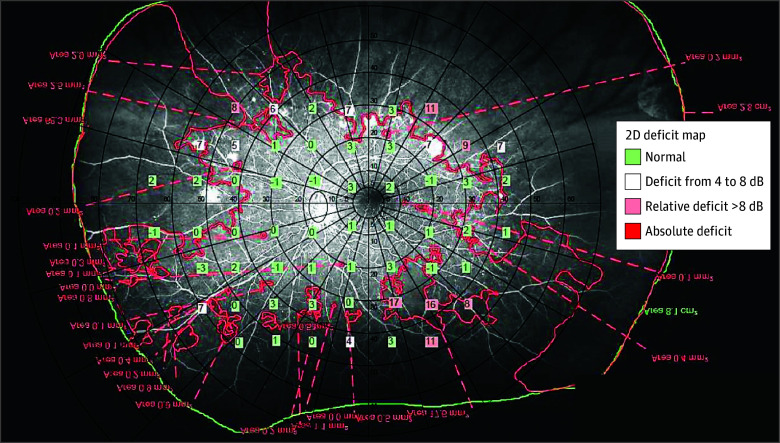
Superimposition of a Perimetric Retinal Sensitivity Map on a Graded Ultra-Widefield Fundus Fluorescein Angiogram of a Participant’s Left Retina The entire retinal area imaged was demarcated in green, while red areas indicate areas of nonperfusion. Green squares indicate normal sensitivity compared with age-matched healthy controls at each particular location, while shades of red indicate areas of reduced sensitivity, with darker shades indicating more significant sensitivity deficits. 2D indicates 2-dimensional.

Perimetric retinal threshold sensitivity values for each eye were superimposed on corresponding graded UWF-FFAs, with fovea and optic disc center as reference points for accurate alignment, ensuring identical locations were compared during follow-up (Figure 1).

Retinal sensitivity values were manually entered into a preformatted grid (eFigure 2 in Supplement 1) to record sensitivity values for each point in their own location and perfusion status (0 = perfused, 1 = nonperfused, and 2 = ungradable), so that pointwise comparisons of sensitivity deficit–perfusion status of identical retinal locations at baseline and follow-up could be made.

Overlapping of sensitivity values on UWF-FFAs was not undertaken until all angiograms had been graded to avoid bias; baseline images were completed first, followed by 1- and 2-year images.

Mean retinal sensitivity deficit was calculated for all perfused and nonperfused areas. Global mean retinal sensitivity deficit (ie, in combined perfused and nonperfused areas) in dBs was also obtained. A mean value of 0 dB corresponds to a field with normal sensitivity; negative and positive values indicate above and below average function, respectively. The percentage of fixation and attention losses was recorded for each examination; exams with fixation losses greater than 33% were considered unreliable.^26^

### Statistical Analysis

The association between perfusion status and mean retinal sensitivity deficit was modeled using mixed-effects linear regression, with perfusion grade as the single fixed effect and patient identification as a random effect to account for potential correlations in sensitivity within individuals. Multiple linear regression was used to assess whether other factors (age, gender, HbA_1c_, severity of DR at baseline) were associated with sensitivity deficit. The association between mean retinal sensitivity deficit in perfused and nonperfused areas and duration of diabetes was modeled using mixed-effects linear regression, with duration of diabetes as the single fixed effect and patient identification as a random effect. Points with absolute retinal sensitivity defects (32 dB) in perfused areas and those with normal sensitivity (defined as ≤5 dB sensitivity deficit)^27,28^ in nonperfused areas at baseline were qualitatively scrutinized (ie, UWF-FFAs reviewed) for potential reasons to explain these findings.

In participants with perimetric examinations at 1 year and those with examinations at both 1 and 2 years, the association between perfusion status and retinal sensitivity deficit over time was evaluated using mixed-effects linear regression, with change in perfusion status and time point as fixed effects and patient identification as a random effect. An interaction term, grade change time point, was included to allow the rate of change in sensitivity to vary by perfusion status.

The association between RII and global mean retinal sensitivity deficit was modeled using linear regression, with RII as the single predictor. We investigated also whether changes in RII over time were associated with percentage gradable area using linear regression, combining data (change in RII from baseline to 1 year and 2 years) in a single model. The reproducibility of RII was evaluated using intraclass correlation coefficient.

Patterns of retinal sensitivity were unique to each eye or time point, with a maximum of 3 perimetric tests per eye. Therefore, we did not consider it appropriate to impute measurements missing due to loss to follow-up and instead conducted complete-case analysis.

A sensitivity analysis was performed, excluding perimetric examinations with attention losses greater than 33%.^26^ *P* values were 2-tailed, with *P* < .05 considered significant. R version 4.2.1 (R Foundation) was used for statistical analyses.

## Results

Of 66 patients invited, 50 accepted and were recruited. All underwent UWF-FFA; 44 had at least 1 perimetric examination. Baseline characteristics of the latter (n = 44) and of those in whom perimetry was not performed (n = 6) are shown in eTable 1 in Supplement 1. Briefly, mean (SD) participant age was 52.1 (12.2) years, and 13 participants (29%) were female. Median hemoglobin A_1c_ was 75.5 mmol/mol (9.1% of total hemoglobin [to convert from percentage of total hemoglobin to proportion of total hemoglobin, multiply by 0.01]); mean (SD) best-corrected visual acuity letter score was 85.7 (4.7) (Snellen equivalent, 20/20).

Retinal area imaged (full area including artifacts), gradable area (without artifacts), total nonperfused area, and RII are shown in eTable 2 in Supplement 1. There were 2508 points—62 were ungradable; hence, 2446 points were studied. All eyes (N = 44) had retinal nonperfusion at baseline; 34 (77%) had measures of retinal sensitivity in areas of CNP. Across all eyes, a mean (SD) of 3.2% (5.5%) of the areas of perfusion or nonperfusion imaged were ungradable.

### Baseline Analysis: Point-to-Point Evaluation

**Figure 2.**
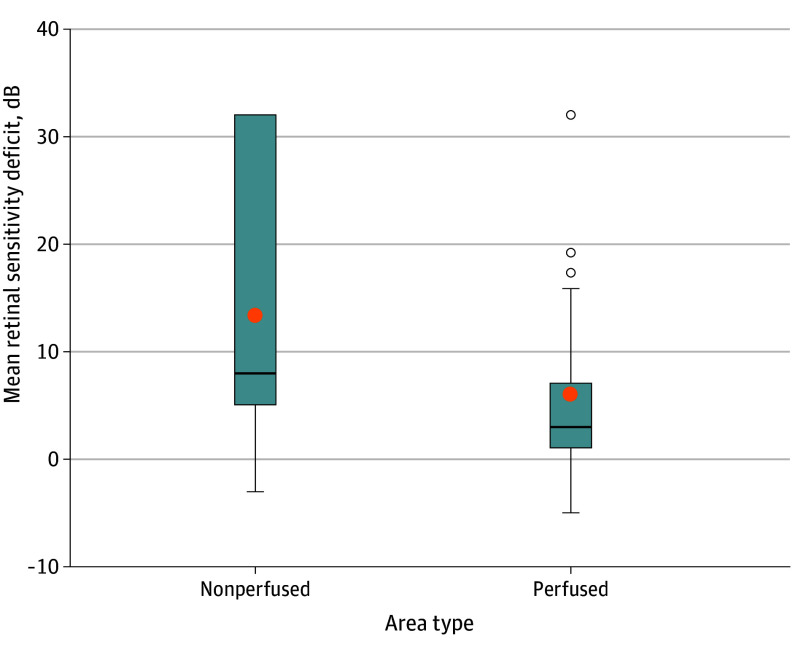
Box Plot Diagram Showing the Retinal Sensitivity Deficit Distribution at 2446 Retinal Areas Tested in 44 Eyes (44 Patients) by Perfusion Status (ie, Perfused and Nonperfused) Box indicates lower quartile, median, and upper quartile; orange dots indicate mean values. *P* value for the comparison between perfused and nonperfused <.005.

There was evidence of an association between retinal sensitivity and perfusion status, with larger retinal sensitivity deficits in nonperfused areas (354 points; 11.8 dB; 95% CI, 10.8-12.8) compared with perfused areas (2092 points; 6.6 dB; 95% CI, 5.1-8.2; *P* < .001) (N = 44 eyes/patients) (Figure 2).

Only age was positively correlated with sensitivity deficit (ie, increasing sensitivity deficit with increasing age) (estimate, 0.2; 95% CI, 0.1-0.3; *P* = .006) (eTable 3 in Supplement 1), with no evidence for an association between retinal sensitivity deficit and duration of diabetes (eTable 4 in Supplement 1). Of points in perfused retina areas, 711 of 2092 (34%) had sensitivity deficits of greater than 5 dB; 105 of 354 (30%) in nonperfused retina had normal sensitivity (Table 1). There were no eyes with normal sensitivity in all areas studied (whether perfused or nonperfused).

**Table 1.** Retinal Sensitivity Deficit by Perfusion Status at Baseline

Perfusion status	No./total No. (%)		
	Perfusion status		Total
	No (deficit ≤5 dB)	Yes (deficit >5 dB)	
Perfused	1381/2092 (66)	711/2092 (34)	2092/2508 (83)
Nonperfused	105/354 (30)	249/354 (70)	354/2508 (14)
Ungradable	38/62 (61)	24/62 (39)	62/2508 (2)
Total	1524/2508 (61)	984/2508 (39)	2508/2508 (100)

Of all points with an absolute scotoma (32 dB) in perfused retina at baseline (190 of 2092 [9%] from 30 of 44 eyes), 105 of 190 (55%) were at sites of blood vessels or adjacent to nonperfused retina, while 85 of 190 (45%) could not be explained other than by altered retinal function. Of all points in nonperfused retina with normal sensitivity at baseline (105 of 354 [30%] from 29 of 44 eyes), 59 of 105 (56%) had a blood vessel traversing the area of nonperfusion, while 46 of 105 (44%) could not be explained other than by having normal function.

#### Follow-Up Analysis: Point-to-Point Evaluation

A total of 27 participants had perimetric examinations at baseline and 1 year (1464 points studied); 10 had it at all time points (542 points studied) (eTable 5 in Supplement 1). Most perfused areas remained perfused (1250 of 1287 [97.1%] at 1 year; 485 of 500 [97.0%] at 2 years); most nonperfused areas remained nonperfused (173 of 177 [97.7%] at 1 year; 39 of 42 [92.9%] at 2 years) (Table 2).

**Table 2.** Changes in Perfusion Status in Retinal Areas From Baseline to 1 Year in 27 Participants (1464 Perimetric Points Studied) and From Baseline to 2 Years in 10 Participants (542 Perimetric Points Studied) With All Follow-Up Visits (Baseline, 1 Year, and 2 Years)

Grade change	Points, No./total No. (%)	
	1 y	2 y
Perfused: perfused	1250/1464 (85.4)	485/542 (89.5)
Perfused: nonperfused	37/1464 (2.5)	15/542 (2.8)
Nonperfused: perfused	4/1464 (0.3)	3/542 (0.6)
Nonperfused: nonperfused	173/1464 (11.8)	39/542 (7.2)
Total	1464/1464 (100)	542/542 (100)

The mean rate of change in sensitivity deficit (less deficit) over time was statistically significant (Table 3). It was not different between perfused and nonperfused areas from baseline to 1 year, but it was different from baseline to 2 years (Table 3). Few perfused areas became nonperfused during follow-up (Table 2), all from 4 eyes/patients. The rate of change in sensitivity deficit in this group (−0.03 dB/month; 95% CI, −0.24 to 0.17) did not differ from that of other groups.

**Table 3.** Mean Rate of Change in Retinal Sensitivity Deficit From Baseline to 1 Year and From Baseline to 2 Years in Areas Classified as Perfused and Nonperfused at Baseline that Remained Perfused and Nonperfused, Respectively, at 1 and 2 Years Follow-Up

Perfusion status at baseline	Baseline to 1 y, dB/mo (95% CI)	*P* value^a^	Baseline to 2 y, dB/mo (95% CI)	*P* value^a^
Eyes, No.	27	NA	10	NA
Perfused	−0.20 (−0.24 to −0.16)	.22	−0.16 (−0.20 to −0.12)	.007
Points, No.	1250		485	
Nonperfused	−0.28 (−0.41 to −0.15)		−0.34 (−0.47 to −0.21)	
Points, No.	173		39	

Abbreviation: NA, not applicable.

^a^ *P* values compare rates in perfused vs nonperfused areas.

#### Baseline Analysis: Global Measures

The mean (SD) RII was 21.0% (17.3%); the mean (SD) global retinal sensitivity deficit was 5.5 dB (3.3) (44 eyes/patients). There was an association between RII and global mean retinal sensitivity deficit. The association between global mean retinal sensitivity deficit (in dB) and RII (percentage) is shown in eFigure 3 in Supplement 1. A positive association between global mean retinal sensitivity deficit and age was found (eTable 6 in Supplement 1).

#### Follow-Up: Global Measures

There were changes in global retinal sensitivity deficit from baseline to 1 year (−1.7 dB; 95% CI, −2.53 to −0.88; paired *t* test, df = 26; *t* = −4.25; *P* < .001) and to 2 years (−2.8 dB; 95% CI, −4.12 to −1.52; *t* = −4.92; df = 9; *P* < .001). There was no evidence for a change in mean RII from baseline to 1 year (1.6%; 95% CI, −0.9% to 4.1%; *t* = 1.28; df = 26; *P* = .21) and to 2 years (0.8%; 95% CI, −3.0% to 4.7%; *t* = 0.48; df = 9; *P* = .64).

Gradable areas varied across time points (mean [SD] percentage change across time points and eyes: −1.1 [9.79]), with no association between magnitude of changes in gradable area and changes in RII (slope coefficient, 0.057; *P* = .58) (eFigure 4 in Supplement 1).

Measurements of RII were highly reproducible (intraclass correlation coefficient, 0.95; 95% CI, 0.87-0.99)

#### Sensitivity Analysis

Thirteen of 83 perimetric examinations (15.7%) (716 points) had attention losses of greater than 33%; sensitivity analyses excluding them (only 3 of 44 eyes were completely excluded, having no valid baseline measurements) did not appear to change the above results, with the following exceptions: there was no longer an association between RII and global mean retinal sensitivity, neither in the difference between perfused and nonperfused areas in rate of sensitivity change between baseline and 2 years.

## Discussion

We found an association between retinal sensitivity deficit, measured using projection perimetry across 110°, and perfusion status throughout the retina, as determined with UWF-FFA, with larger (79%) deficits and wider variability in retinal function in nonperfused than perfused retinal areas. Marked functional loss was detected in approximately one-third of perfused areas, and normal function was found in a similar proportion of nonperfused areas. Only age seemed to modulate retinal sensitivity. The perfusion status of most retinal points did not change. Considerable changes in retinal sensitivity in perfused and nonperfused areas occurred during the follow-up of up to 2 years, with lesser sensitivity deficits observed over time. The RII was positively correlated with mean retinal sensitivity deficit and age, although the former was not robust to sensitivity analysis. A reduction in global sensitivity deficit was observed over time.

Altered function in perfused retina might be explained by metabolic factors,^8^ established neurodegeneration, or deep capillary plexus dropout, not detected with FFA in studies using this technology. Against the latter is the finding of decreased superficial, but not deep, capillary vessel density on OCTA being associated with worsening of sensitivity at the fovea over 1 year.^19^ We observed that around half of the points with reduced sensitivity in perfused retina were at sites of blood vessels and/or close to nonperfused retina. Thus, sensitivity loss could relate to the lack of sensitivity over blood vessels and/or dysfunction at the penumbra. This highlights the importance of meticulous evaluations in studies of this sort to interpret findings.

Bek suggested that the presence of normal sensitivity at sites of nonperfusion could be explained by direct diffusion of oxygen or nutrients from perfused vessels crossing these areas.^11^ We found this could be the case in more than half of the nonperfused retinal areas with normal sensitivity. In the remaining areas, vasodegeneration may precede functional loss. It is also conceivable that retinal function may be maintained by diffusion of oxygen or nutrients from adjacent perfused retina, especially if areas of nonperfusion are small^9^ or from the choroid,^29^ although this is less likely, considering that choroidal ischemia is also prominent in DR.^30^

Our study found sizable changes in retinal sensitivity over time. The reduction in retinal sensitivity deficit over time needs to be considered when designing clinical trials evaluating new therapies for retinal CNP if this were to be included as an outcome measure. Improvements in global perimetric measures over time of approximately 1 to 3 dB have been reported in some,^31,32,33^ but not all,^34,35^ previous studies in a proportion of healthy and glaucomatous participants when repeating visual fields at short intervals,^31,32,36,37^ and have been attributed to learning effects. Like in our study, larger improvements over time were detected in areas with larger baseline deficits.^31,32,33^ Larger improvements were also described with increased eccentricity from the fovea.^31,32^ Whether all improvement in sensitivity could be attributed to learning effects is currently unknown. It may be possible that the chronic nature of DR allows compensatory reparative mechanisms to be activated that may account for a degree of functional recovery over time.

### Strengths and Limitations

Strengths of our study include its prospective and longitudinal design, the relatively long follow-up, the use of UWF-FFA and full-field projection perimetry to facilitate detailed point-to-point correlations, and the high number of retinal areas studied. All perimetric examinations were performed by the same examiner under identical test conditions. The same retinal points were assessed perimetrically at baseline and follow-up, as tracked by the device. UWF-FFAs were graded masked to clinical and perimetric findings. Changes in retinal sensitivity and perfusion were assessed for each participant at each time point (ie, comparing each participant with him-, her-, or themself). A sensitivity analysis was conducted, omitting possible unreliable perimetric tests. Findings were scrutinized by evaluating images meticulously to aid their interpretation. Limitations include the small cohort of participants, especially at 2 years. Grading of retinal perfusion may be considered subjective, with intrinsic limitations. Ungradable areas could not be analyzed.

## Conclusions

This longitudinal cohort study demonstrates that retinal ischaemia in DR is complex, and its impact on retinal function is nonuniform. Methodological scrutiny and meticulosity when undertaking research on this area are essential.

## Notes

Supplement 1. eFigure 1. Diabetic Retinopathy Severity Classification Scheme Used in the Study eFigure 2. Grading Grid to Record Sensitivity Values for Each Point and Corresponding Perfusion Status (0=Perfused, 1=Nonperfused, 2=Ungradable), Enabling Precise Pointwise Statistical Comparisons of Sensitivity Deficit-Perfusion Status at Identical Retinal Locations at Baseline and Follow-Up eFigure 3. Scatterplot Showing the Relationship Between Global Mean Retinal Sensitivity Deficit (dB) and Retinal Ischaemic Index (%) at Baseline eFigure 4. Change in Retinal Ischaemic Index vs. Percentage Change in Gradable Area eTable 1. Baseline Characteristics eTable 2. Retinal Areas Analysed (Imaged, Graded, and Nonperfused), Retinal Ischaemic Index and Number of Retinal Sensitivity Points Studied at Baseline eTable 3. Multivariable Regression Analysis Evaluating Variables Potentially Associated With Retinal Sensitivity Deficit (dB) eTable 4. Estimated Associations Between Retinal Sensitivity Deficit (dB) and Duration of Diabetes (Years) in Perfused and Nonperfused Areas eTable 5. Comparison of the Characteristics of Participants Who Had and Did Not Have Perimetry at 1- and 2-Year Follow-Up eTable 6. Multiple Regression Analysis Model Evaluating Associations Between Global Mean Retinal Sensitivity Deficit (dB) and Retinal Ischaemic Index (%)

Supplement 2. Data Sharing Statement
