# Supplementary material for: Retinal Sensitivity and Retinal Perfusion in Diabetic Retinopathy
Source: JAMA Ophthalmol. 2025 Oct 30;144(1):91–7. doi: 10.1001/jamaophthalmol.2025.3980 (PMC12576616; doi:10.1001/jamaophthalmol.2025.3980)
Supplement: Supplement 1. — eFigure 1. Diabetic Retinopathy Severity Classification Scheme Used in the Study eFigure 2. Grading Grid to Record Sensitivity Values for Each Point and Corresponding Perfusion Status (0=Perfused, 1=Nonperfused, 2=Ungradable), Enabling Precise Pointwise Statistical Comparisons of Sensitivity Deficit-Perfusion Status at Identical Retinal Locations at Baseline and Follow-Up eFigure 3. Scatterplot Showing the Relationship Between Global Mean Retinal Sensitivity Deficit (dB) and Retinal Ischaemic Index (%) at Baseline eFigure 4. Change in Retinal Ischaemic Index vs. Percentage Change in Gradable Area eTable 1. Baseline Characteristics eTable 2. Retinal Areas Analysed (Imaged, Graded, and Nonperfused), Retinal Ischaemic Index and Number of Retinal Sensitivity Points Studied at Baseline eTable 3. Multivariable Regression Analysis Evaluating Variables Potentially Associated With Retinal Sensitivity Deficit (dB) eTable 4. Estimated Associations Between Retinal Sensitivity Deficit (dB) and Duration of Diabetes (Years) in Perfused and Nonperfused Areas eTable 5. Comparison of the Characteristics of Participants Who Had and Did Not Have Perimetry at 1- and 2-Year Follow-Up eTable 6. Multiple Regression Analysis Model Evaluating Associations Between Global Mean Retinal Sensitivity Deficit (dB) and Retinal Ischaemic Index (%) [file jamaophthalmol-e253980-s001.pdf]

Supplemental Online Content

Hamilton-Perais JA, Wright D, Lim A, et al. Retinal sensitivity and retinal perfusion in diabetic retinopathy. *JAMA Ophthalmol*. Published online October 30, 2025. doi:10.1001/jamaophthalmol.2025.3980

eFigure 1. Diabetic Retinopathy Severity Classification Scheme Used in the Study

eFigure 2. Grading Grid to Record Sensitivity Values for Each Point and Corresponding Perfusion Status (0=Perfused, 1=Nonperfused, 2=Ungradable), Enabling Precise Pointwise Statistical Comparisons of Sensitivity Deficit-Perfusion Status at Identical Retinal Locations at Baseline and Follow-Up

eFigure 3. Scatterplot Showing the Relationship Between Global Mean Retinal Sensitivity Deficit (dB) and Retinal Ischaemic Index (%) at Baseline

eFigure 4. Change in Retinal Ischaemic Index vs. Percentage Change in Gradable Area

eTable 1. Baseline Characteristics

eTable 2. Retinal Areas Analysed (Imaged, Graded, and Nonperfused), Retinal Ischaemic Index and Number of Retinal Sensitivity Points Studied at Baseline

eTable 3. Multivariable Regression Analysis Evaluating Variables Potentially Associated With Retinal Sensitivity Deficit (dB)

eTable 4. Estimated Associations Between Retinal Sensitivity Deficit (dB) and Duration of Diabetes (Years) in Perfused and Nonperfused Areas

eTable 5. Comparison of the Characteristics of Participants Who Had and Did Not Have Perimetry at 1- and 2-Year Follow-Up

eTable 6. Multiple Regression Analysis Model Evaluating Associations Between Global Mean Retinal Sensitivity Deficit (dB) and Retinal Ischaemic Index (%)

This supplemental material has been provided by the authors to give readers additional information about their work.

© 2025 Hamilton-Perais JA et al. *JAMA Ophthalmology*.

eFigure 1. Diabetic Retinopathy Severity Classification Scheme Used in the Study

Moderate NPDR

More than "mild" NPDR (=microaneurysms only).

Severe/very severe NPDR

Fulfilling one or more of the 4-2-1 rule (severe hemorrhages in all four quadrants; venous beading in 2 or more quadrants; IRMA in 1 or more quadrants).

PDR less than HRC

Presence of new vessels in the disc or elsewhere in the retina (confirmed on ultrawide field fluorescein angiography) but no HRC (HRC = NVD greater than 1/4 to 1/3 disc area, presence of preretinal or vitreous hemorrhage).

NPDR = Non-proliferative diabetic retinopathy; IRMA = Intraretinal microvascular abnormalities; PDR = Proliferative diabetic retinopathy; HRC = High-risk characteristics; NVD = New vessels at the disc.

© 2025 Hamilton-Perais JA et al. *JAMA Ophthalmology*.

eFigure 2. Grading Grid to Record Sensitivity Values for Each Point and Corresponding Perfusion Status (0=Perfused, 1=Nonperfused, 2=Ungradable), Enabling Precise Pointwise Statistical Comparisons of Sensitivity Deficit-Perfusion Status at Identical Retinal Locations at Baseline and Follow-Up

IDR .... Date .... Visit .... RIGHT EYE

	1	2	3	4	5	6	7	8	9	10
A										
B										
C										
D										
E										
F										
G										

IDR .... Date .... Visit .... LEFT EYE

	10	9	8	7	6	5	4	3	2	1
A										
B										
C										
D										
E										
F										
G										

© 2025 Hamilton-Perais JA et al. *JAMA Ophthalmology*.

eFigure 3. Scatterplot Showing the Relationship Between Global Mean Retinal Sensitivity Deficit (dB) and Retinal Ischaemic Index (%) at Baseline

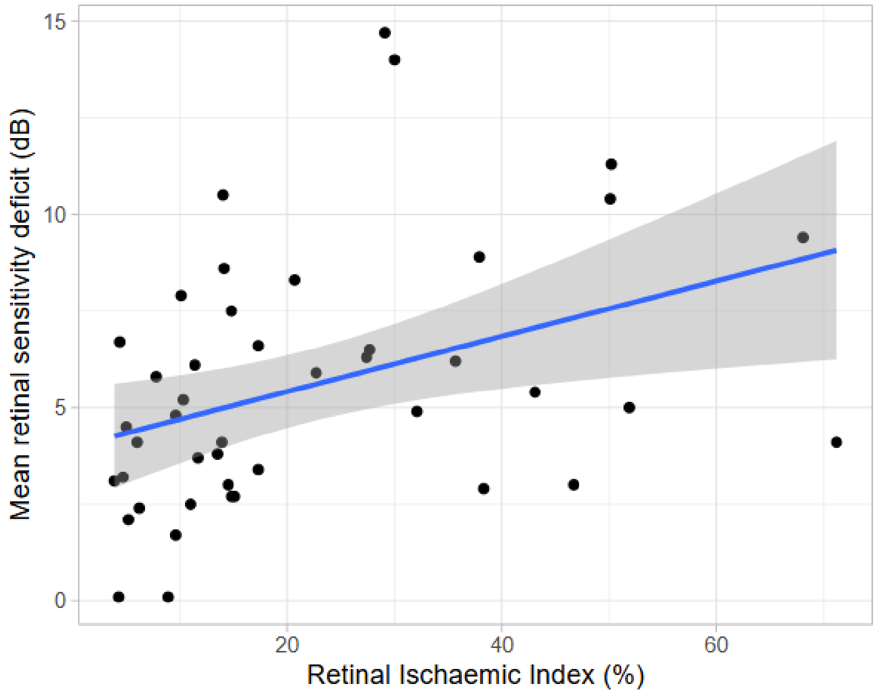

Linear regression line and 95% confidence region shown.

© 2025 Hamilton-Perais JA et al. *JAMA Ophthalmology*.

eFigure 4. Change in Retinal Ischaemic Index vs. Percentage Change in Gradable Area

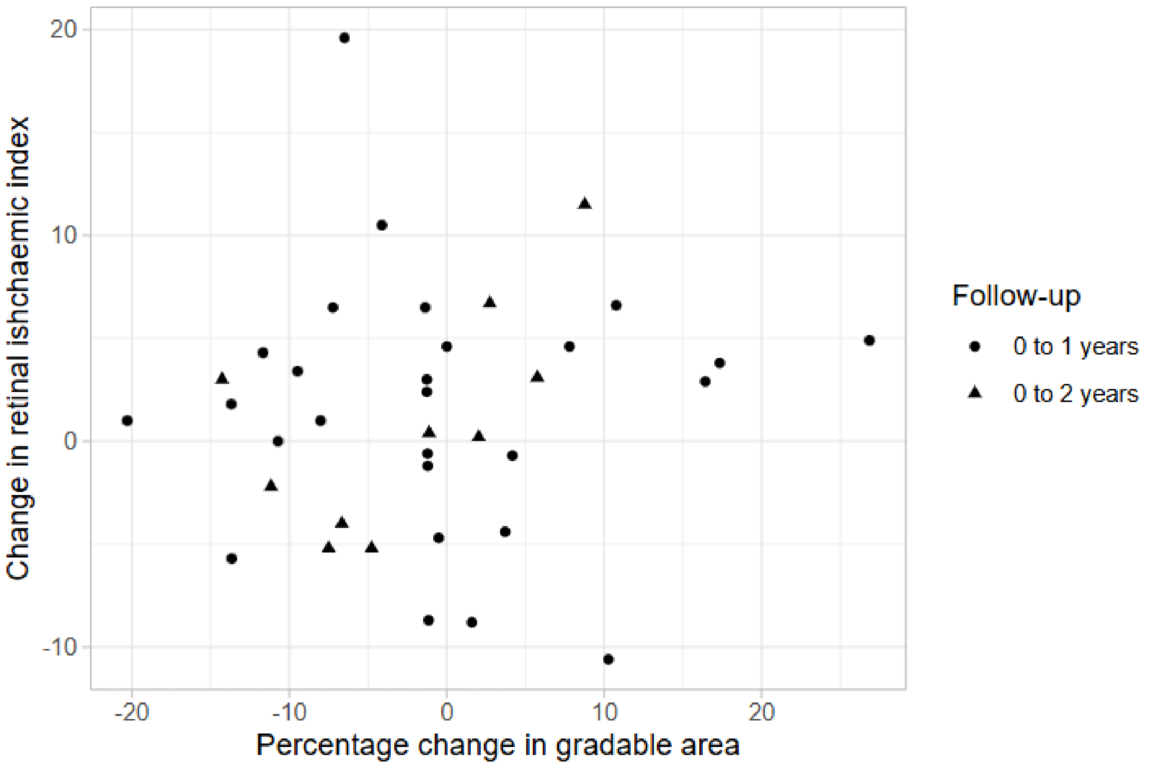

© 2025 Hamilton-Perais JA et al. *JAMA Ophthalmology*.

eTable 1. Baseline Characteristics

**Baseline characteristics**	**Cohort of patients who had perimetry (n=44)**	**Cohort of patients who did not have perimetry [excluded] (n=6)**
Age (years), Mean ± SD	52.1 ± 12.2	53.8 ± 9.4
Sex, male, n (%)	31 (71%)	5 (83%)
Type of diabetes, type 1, n (%)	25 (56.8%)	4 (67%)
Duration of diabetes, mean ± SD	20.5 ± 8.9	19.5 ± 7.6
Diabetic retinopathy grading, n (%)		
Moderate non-proliferative	23 (52.3%)	2 (33%)
Severe-very severe non-proliferative	10 (22.7%)	0 (0%)
Proliferative < high-risk characteristics	11 (25.0%)	4 (67%)
Visual acuity (ETDRS letters), mean ± SD	85.7 ± 4.7	84.5 ± 8.2
HbA1c (mmol/mol)[a], mean ± SD	75.3 ± 20.0	84.7 ± 14.9
HbA1c (mmol/mol), median, range	75.5, 48-133	89.5, 57-97
Body mass index[b], mean ± SD	29.6 ± 5.8	31.56 ± 4.8[c]

n = number; SD = standard deviation; ETDRS = Early Treatment Diabetic Retinopathy Study; HbA1c = glycated haemoglobin; mmol/mol = millimoles per mole; [a] Expected normal value: 23.5 – 43.2; [b] Expected normal value: 18 – 24.9; [c] measurement available for 5 of the 6 participants.

© 2025 Hamilton-Perais JA et al. *JAMA Ophthalmology*.

eTable 2. Retinal Areas Analysed (Imaged, Graded, and Nonperfused), Retinal Ischaemic Index and Number of Retinal Sensitivity Points Studied at Baseline

**Measure at baseline (n=44 eyes)**	**Mean**	**SD**	**Median**	**Interquartile range**
Retinal area imaged mm^2 (SD)	788.5	59.1	790.0	760.0-830.0
Retinal area graded (retinal area imaged – total area of artefacts) mm^2 (SD)	756.3	67.4	770.0	725.4-809.5
Nonperfused area mm^2 (SD)	161.8	137.3	110.0	76.1-239.8
Retinal ischaemic index % (SD)	21.1	16.8	14.7	9.6-30.5
Total number of perimetric retinal points evaluated	2446			

n = number; mm = millimetre; SD = standard deviation

© 2025 Hamilton-Perais JA et al. *JAMA Ophthalmology*.

eTable 3. Multivariable Regression Analysis Evaluating Variables Potentially Associated With Retinal Sensitivity Deficit (dB)

**Term**	**Estimate**	**95% lower confidence interval**	**95% upper confidence interval**	***P* value**
Age	0.17	0.05	0.29	.006
Sex	0.78	-2.35	3.90	.62
DR severity at baseline: PDR<HRC	-1.20	-4.24	1.85	.43
HbA1c (mmol/mol)	0.07	-0.01	0.14	.07
Nonperfused	5.19	4.16	6.21	<.001

n = number; DR = diabetic retinopathy; PDR<HRC = proliferative diabetic retinopathy with less than high-risk characteristics; HbA1c = glycated haemoglobin; mmol/mol = millimoles per mole.

© 2025 Hamilton-Perais JA et al. *JAMA Ophthalmology*.

eTable 4. Estimated Associations Between Retinal Sensitivity Deficit (dB) and Duration of Diabetes (Years) in Perfused and Nonperfused Areas

**Perfusion status**	**Term**	**Estimate**	**95% lower confidence interval**	**95% upper confidence interval**	***P* value**
Perfused	Duration of diabetes (years)	0.02	-0.17	0.20	.85
Noperfused	Duration of diabetes (years)	0.10	-0.21	0.41	.52

© 2025 Hamilton-Perais JA et al. *JAMA Ophthalmology*.

eTable 5. Comparison of the Characteristics of Participants Who Had and Did Not Have Perimetry at 1- and 2-Year Follow-Up

**Characteristics**	**One Year**		**Two Year**	
	**Perimetry (n=27)**	**No perimetry (n=17)**	**Perimetry (n=10)**	**No perimetry (n=34)**
Age (years), Mean ± SD	49.5 ± 13.9	56.1 ± 7.5	55.4 ± 13.6	51.1 ± 11.8
Sex, male, n (%)	18 (67%)	13 (76%)	8 (80%)	23 (68%)
Type of diabetes, type 1, n (%)	17 (63.0%)	12 (70.6%)	7 (70%)	23 (67.6%)
Duration of diabetes, mean ± SD	20.6 ± 9.5	20.1 ± 8.0	23.2 ± 8.7	19.6 ± 8.9
Diabetic retinopathy grading, n (%)				
Moderate non-proliferative	15 (55.6%)	7 (41.2%)	8 (80.0%)	14 (41.2%)
Severe-very severe non-proliferative	5 (18.5%)	4 (23.5%)	2 (20.0%)	7 (20.6%)
Proliferative < high risk characteristics	7 (25.9%)	6 (35.3%)	0 (0.0%)	13 (38.2%)
Visual acuity (ETDRS letters), mean ± SD	86.5 ± 4.8	84.7 ± 4.3	87.4 ± 3.9	85.3 ± 4.8
HbA1c (mmol/mol)[a], mean ± SD	72.1 ± 16.4	83.1 ± 23.5	69.8 ± 18.9	78.2 ± 20.0
HbA1c (mmol/mol), median, range	67, 50-118	82, 48-133	65, 50-118	78, 48-133
Body mass index[b], mean ± SD	28.7 ± 6.1	31.0 ± 5.1	28.3 ± 5.7	30.0 ± 5.8

n = number; SD = standard deviation; ETDRS = Early Treatment Diabetic Retinopathy Study; HbA1c = glycated haemoglobin; mmol/mol = millimoles per mole; [a] Expected normal value: 23.5 – 43.2; [b] Expected normal value: 18 – 24.9; [c] measurement available for 5 of the 6 participants.

© 2025 Hamilton-Perais JA et al. *JAMA Ophthalmology*.

eTable 6. Multiple Regression Analysis Model Evaluating Associations Between Global Mean Retinal Sensitivity Deficit (dB) and Retinal Ischaemic Index (%)

**Term**	**Estimate**	**95% lower confidence interval**	**95% upper confidence interval**	***P* value**
Retinal ischaemic index (%)	0.06	0.01	0.12	.03
Age	0.09	0.02	0.17	.015
Sex (0=female; 1=male)	0.26	-1.89	2.41	.81
DR severity at baseline: PDR<HRC	-0.19	-2.14	1.77	.85
HbA1c (mmol/mol)	0.04	-0.01	0.08	.11

© 2025 Hamilton-Perais JA et al. *JAMA Ophthalmology*.
