# Supplementary material for: Retinal Sensitivity and Retinal Perfusion in Diabetic Retinopathy
Source: JAMA Ophthalmol. 2025 Oct 30;144(1):91–7. doi: 10.1001/jamaophthalmol.2025.3980 (PMC12576616; doi:10.1001/jamaophthalmol.2025.3980)
Supplement: Supplement 2. — Data Sharing Statement [file jamaophthalmol-e253980-s002.pdf]

Data Sharing Statement

Hamilton-Perais. Retinal Sensitivity and Retinal Perfusion in Diabetic Retinopathy. *JAMA Ophthalmol*. Published October 30, 2025. doi:10.1001/jamaophthalmol.2025.3980

Data
Data available: Yes
Data types: Other (please specify)
Additional Information: Data from this study will be shared upon reasonable request after discussion with the corresponding author (CI).
How to access data: Anonymised data. n.lois@qub.ac.uk
When available: beginning date: 09-01-2026

Supporting Documents
Document types: None

Additional Information
Who can access the data: Researchers.
Types of analyses: The type of analyses will depend on the research question(s).
Mechanisms of data availability: With support of the investigator providing that funds are available.
